# Research on Gate Opening Control Based on Improved Beetle Antennae Search

**DOI:** 10.3390/s24134425

**Published:** 2024-07-08

**Authors:** Lijun Wang, Yibo Wang, Yehao Kang, Jie Shen, Ruixue Cheng, Jianyong Zhang, Shuheng Shi

**Affiliations:** 1 School of Mechanical Engineering, North China University of Water Resources and Electric Power, Zhengzhou 450045, China; 201621104@stu.ncwu.edu.cn (Y.W.); kangyh5928@163.com (Y.K.); shenjie@ncwu.edu.cn (J.S.); 2 School of Computing, Engineering and Digital Technologies, Teesside University, Middlesbrough TS1 3BX, UK; r.cheng@tees.ac.uk (R.C.); j.zhang@tees.ac.uk (J.Z.)

**Keywords:** beetle antennae search algorithm, control system, brushless DC motor, gate control system, PID control

## Abstract

To address the issues of sluggish response and inadequate precision in traditional gate opening control systems, this study presents a novel approach for direct current (DC) motor control utilizing an enhanced beetle antennae search (BAS) algorithm to fine-tune the parameters of a fuzzy proportional integral derivative (PID) controller. Initially, the mathematical model of the DC motor drive system is formulated. Subsequently, employing a search algorithm, the three parameters of the PID controller are optimized in accordance with the control requirements. Next, software simulation is employed to analyze the system’s response time and overshoot. Furthermore, a comparative analysis is conducted between fuzzy PID control based on the improved beetle antennae search algorithm, and conventional approaches such as the traditional beetle antennae search algorithm, the traditional particle swarm algorithm, and the enhanced particle swarm algorithm. The findings indicate the superior performance of the proposed method, characterized by reduced oscillations and accelerated convergence compared to the alternative methods.

## 1. Introduction

Water conservancy serves as the lifeblood of the national economy, with gate stations representing pivotal water conservancy infrastructure. These projects shoulder critical responsibilities for basin and regional flood control, drainage, irrigation, and water transfer. They play an indispensable role in managing floods, mitigating droughts, and effectively managing water resources. Furthermore, they serve as vital sources for industrial and agricultural water supplies, as well as urban and rural water supplies, underscoring their significant societal importance.

Direct current (DC) motors exhibit commendable performance in terms of starting and speed regulation, making them a preferred choice for applications with stringent requirements. They find extensive usage in scenarios such as winch-type openers, ship machinery, large precision machine tools, and heavy-duty cranes, among other industrial machinery. Consequently, in water conservancy gate systems, the DC motor is often favored as the primary gate opener. By simplifying the gate opening control to control the DC motor, it is imperative for the motor to achieve stable and precise control parameters [1].

The proportional integral derivative (PID) control scheme used in traditional motor control systems is most widely used in industrial production due to its simple control structure, strong robustness, high accuracy, etc. The main function of the PID controller is to regulate the feedback signal to be as similar as possible to the input signal, and a faster response speed and higher accuracy can be obtained by choosing the appropriate PID parameters. However, with the advancement of science and technology and the improvement of accuracy requirements, classical PID control technology has increasingly shown that signal processing is too simple and that the control parameters are not adjusted due to the environment and other shortcomings; therefore, the PID parameter tuning method has become a popular research topic.

With the development of artificial intelligence, artificial intelligence algorithms, such as the particle swarm optimization algorithm, are widely used in PID controllers to select reasonable PID parameters [2]. The beetle antennae search algorithm is an efficient intelligent search algorithm, proposed by Jiang Xiangyuan and Li Shuai in 2017. It is a genetic algorithm derived from the foraging behaviors of beetles. The algorithm is able to recognize individuals and environments without knowing functional information, and the algorithm has simple code implementation, fast computation speed, and the ability to obtain the optimal solution under stable convergence. BAS algorithms have been applied in the fields of neural network optimization [3], electrohydraulic servo position control optimization [4], industrial robot control [5], and unmanned aerial vehicle (UAV) sensing and avoidance [6].

In summary, the optimization of PID parameters by a genetic algorithm still has the disadvantages of requiring a long adjustment time and easily falling into a locally optimal state. Therefore, in this paper, the improved beetle antennae search algorithm is used for the adaptive optimization of fuzzy PID controller parameters so that the parameters can be adaptively adjusted to adapt to load and environmental changes. By optimizing the step size of the beetle antennae search algorithm, the response speed and convergence accuracy of the system are effectively improved, and the step size is changed with the iteration to prevent the algorithm from falling into the local optimal solution, which ensures the speed and accuracy of the algorithm.

In this paper, we use MATLAB/Simulink software to construct a corresponding control system simulation model, simulate the response speed and accuracy, and test and verify the performance of the electric winch gate opener in a real working environment. By monitoring the motor speed when the motor is raised under different optimization algorithms, the simulation and experimental results show that the improved beetle antennae search algorithm has a better effect on fuzzy PID parameter tuning than other algorithms, and the motor control system has a better control effect.

## 2. Modeling of the Brushless DC Motor Control System

### 2.1. Brushless DC Motor System

**Figure 1:**
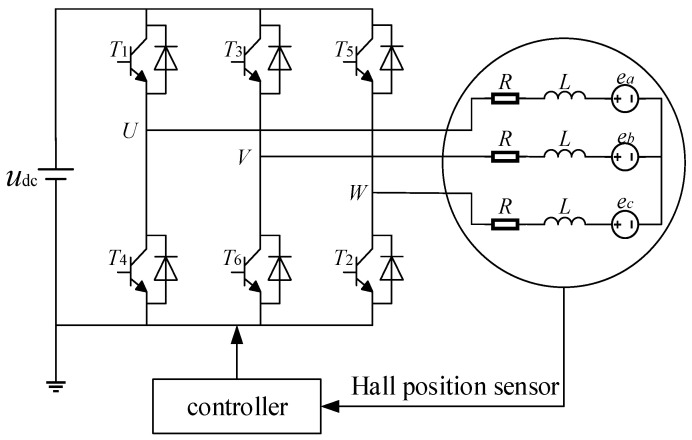
Schematic diagram of the brushless DC motor control system.

According to the schematic diagram of the two-phase conduction star three-phase six-state control system of the brushless DC motor shown in Figure 1, the brushless DC motor (BLDCM) mathematical model is established, and the following assumptions are made to simplify the establishment and analysis of the mathematical model of the motor:

The three-phase windings U, V, and W inside the motor are assumed to be symmetrically and uniformly continuously distributed;

The tube voltage drop across the power tubes and the continuity diode in the inverter of the motor control system are neglected;

The effects of the cogging effect, magnetic circuit saturation, and armature reaction on the motor are neglected;

The effects of hysteresis, eddy current effect, skin effect, and temperature change during operation on the motor parameters are neglected.

Based on the above assumptions, the matrix expression for the stator voltage balance equation of the brushless DC hub motor can be written as

(1) $$\left[\begin{array}{c} u_{a}\\ u_{b}\\ u_{c} \end{array}\right]=\left[\begin{array}{ccc} R & 0 & 0\\ 0 & R & 0\\ 0 & 0 & R \end{array}\right]\left[\begin{array}{c} i_{a}\\ i_{b}\\ i_{c} \end{array}\right]+\left[\begin{array}{ccc} L_{s} & M & M\\ M & L_{s} & M\\ M & M & L_{s} \end{array}\right]\frac{d}{dt}\left[\begin{array}{c} i_{a}\\ i_{b}\\ i_{c} \end{array}\right]+\left[\begin{array}{c} e_{a}\\ e_{b}\\ e_{c} \end{array}\right]$$

where $u_{a}$, $u_{b}$, and $u_{c}$ are the U, V, and W three-phase voltages, V; $i_{a}$, $i_{b}$, and $i_{c}$ are the U, V, and W three-phase currents, A; $e_{a}$, $e_{b}$, and $e_{c}$ are the U, V, and W three-phase opposite electromotive forces, V; R is the motor three-phase winding resistance, Ω; $L_{s}$ is the inductance of the winding, H; and M is the mutual inductance of each of the two phases of the winding before H.

The motor electromagnetic torque expression is

(2) $$T_{e}=(e_{a}i_{a}+e_{b}i_{b}+e_{c}i_{c})/\omega$$

where: $T_{e}$ is the electromagnetic torque of the motor, Nm, and ω is the mechanical angular velocity of the motor when rotating, rad/s.

The equations of equilibrium of motion of the motor rotor are

(3) $$T_{e}-T_{L}=J\frac{d\omega }{dt}+B\omega$$

where: $T_{L}$ is the motor load torque, Nm; *J* is the motor rotor moment of inertia, $\mathrm{kg}\cdot m^{2}$; and *B* is the motor viscous friction factor.

### 2.2. Modeling of a DC Motor Drive Train Considering the Structural Stiffness of the Drive Shaft

The motor is directly coupled to the load, but the drive shaft is inherently elastic and therefore subject to deformation, and the bearings and frame cannot be completely rigid. For systems with large acceleration requirements, high speed and accuracy requirements, or systems with large rotational inertia and high performance requirements, the effect of elastic deformation on the system performance cannot be ignored; therefore, to establish a similar motor-load model, the stiffness coefficient of the shaft, i.e., the torque generated by the unit angle of rotation, is an important parameter.

**Figure 2:**
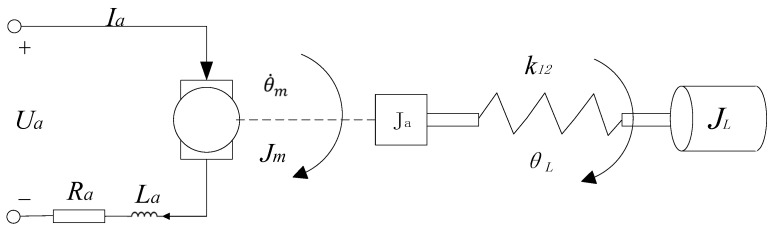
Load model of the DC motor drive mechanism.

Considering the presence of the various elastomers described above, the controlled system can be considered the system structure illustrated in Figure 2: a three-mass system consisting of a motor, a purely inertial load, and an equivalent transfer axis connected to both.

Building upon the aforementioned analysis and disregarding the rotational inertia of the shaft, the electrical equations along with the dynamic equations for the entire system can be formulated as follows:

Electric motors:

(4) $$iR_{a}+L_{a}\frac{di}{dt}=u_{a}-K_{e}{\overset{\cdot }{\theta }}_{m}$$

(5) $$T_{m}=ik_{m}$$

(6) $$J_{m}{\overset{\cdot \cdot }{\theta }}_{m}=T_{m}-D_{m}{\overset{\cdot }{\theta }}_{m}-k_{12}(\theta_{m}-\theta_{L})$$

where: $\theta_{m}$ is the motor rotor; $\theta_{L}$ is the load angle; i is the motor armature current; $U_{a}$ is the motor armature voltage; $L_{a}$ is the motor armature inductance; $R_{a}$ is the motor armature resistance; and $J_{m}$ is the rotational inertia of the motor rotor.

Load:

(7) $$J_{L}{\overset{\cdot \cdot }{\theta }}_{L}=T_{mL}-D_{L}{\overset{\cdot }{\theta }}_{L}$$

(8) $$K_{12}(\theta_{m}-\theta_{L})-T_{mL}=0$$

where: $J_{L}$ is the rotational inertia of the motor rotor; $T_{mL}$ is the load moment of the motor; $K_{m}$ is the electromagnetic torque coefficient of the motor; $K_{e}$ is the reaction potential of the motor; $K_{12}$ is the stiffness coefficient of the shaft; $D_{L}$ is the viscous damping coefficient of the motor; and $D_{m}$ is the viscous damping coefficient of the frame.

**Figure 3:**
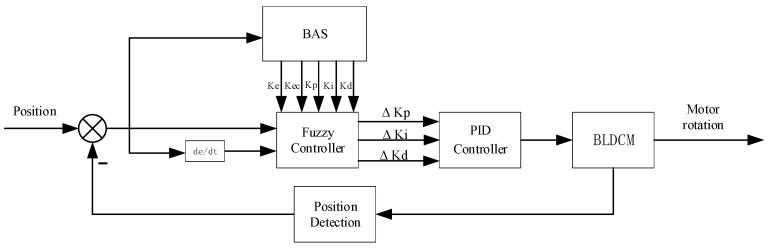
Gate opening control system.

Figure 3 illustrates the structure of the gate opening control system. To enhance the control process efficiency, minimize the corresponding control system time, and optimize the control system accuracy, a control system model for the gate is developed. The control model for the gate control system presented in this paper is based on the preset gate position determined by the system as input, the real-time position acquired through the position sensor as feedback, and the motor-controlled gate opening mechanism.

## 3. Fuzzy Pid Control System Based on an Improved Beetle Antennae Search

### 3.1. Fuzzy PID Control

**Figure 4:**
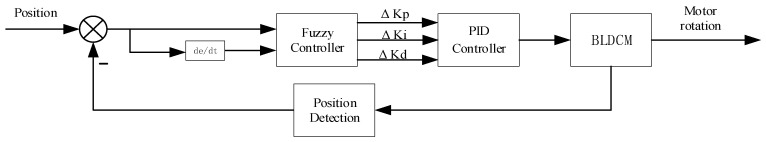
Schematic diagram of fuzzy PID control.

Fuzzy PID control is a method that combines fuzzy logic with PID control, harnessing the strengths of both control strategies. This approach surpasses the constraints of conventional PID control when dealing with nonlinear, time-varying, and intricate systems. Moreover, fuzzy PID control exhibits greater adaptability in application, catering to diverse systems and control requirements. The fuzzy controller employs the error (e) and the rate of change of error (ec) between the preset gate position and the real-time position as input variables. The three stages of fuzzification, fuzzy inference, and defuzzification yield the three parameter variables ${\Delta k}_{p}$, $\Delta k_{i}$, and ${\Delta k}_{d}$, respectively, which enable real-time adjustment of the PID parameters. A block diagram depicting the principle of fuzzy PID control is shown in Figure 4.

The adjusted controller parameters are

(9) $$\left\{\begin{array}{l} k_{p}=k_{p0}+\Delta k_{p}\\ k_{i}=k_{i0}+\Delta k_{i}\\ k_{d}=k_{d0}+\Delta k_{d} \end{array}\right.$$

where: $k_{p}$, $k_{i}$, and $k_{d}$ are the adjusted parameters; $k_{p0}$, $k_{i0}$, and $k_{d0}$ are the three initial PID values; and ${\Delta k}_{p}$, $\Delta k_{i}$, and ${\Delta k}_{d}$ are the output parameter variables of the fuzzy controller.

In fuzzy control, the formulation of fuzzy rules is the cornerstone of a fuzzy controller. Typically, the input signal undergoes fuzzification, followed by fuzzy inference, and ultimately, the signal-derived postfuzzy inference is defuzzified, underscoring the significance of fuzzy rules [7]. A fuzzy rule encompasses a sequence of fuzzy conditions amalgamated to construct a fuzzy database. Within a fuzzy set, *R* denotes the overall set, *i* represents the *i*th subrule, *n* signifies the total number of rules, *Si* denotes the *i*th subset, and the aggregation of all subsets equals the total set *R*, as depicted in Equation (10):

(10) $$R=\overset{n}{\underset{i=1}{\cup }}R_{i}=R_{1}\cup R_{2}\cup •••\cup R_{n-1}\cup R_{n}$$

This system divides the PID deviation e into seven subfuzzy control sets when establishing fuzzy rules: negative big (NB), negative medium (NM), negative small (NS), zero (Z), positive small (PS), positive medium (PM), and positive big (PB). Moreover, the fuzzy rules based on the deviation range of variable e are divided into seven partial value ranges: −3, −2, −1, 0, 1, 2, 3, and $K_{p}/K_{i}/K_{d}$, as shown in Table 1.

The quantization factor and proportionality factor are pivotal parameters utilized to fine-tune the output of fuzzy controllers. They serve to regulate the discrete output level, precision, and gain, thereby facilitating precise system control and ensuring system stability. Initially, deriving these factors entails approximate calculations and accumulated experience, a process that is both time-consuming and challenging.

Fuzzy inference represents a crucial stage within fuzzy control systems, converting fuzzy inputs into fuzzy outputs. During this process, input values are initially mapped to appropriate membership values within the fuzzy set based on system inputs and predefined fuzzy rules. Subsequently, the corresponding fuzzy output values are computed by applying logical relationships inherent in the fuzzy rules. Defuzzification involves mapping the fuzzy output to the actual output space for practical control or decision-making purposes. Commonly employed defuzzification methods include the maximum affiliation method, median method, and center of gravity method. When selecting a method, considerations such as system performance requirements, real-time constraints, complexity, and computational efficiency must be considered. The center of gravity method, notable for its intuitiveness and simplicity in calculation, offers resolution and clear physical interpretation. Therefore, this paper adopts the center of gravity method for defuzzification purposes [8]. The center of gravity method is described by the following equation:

(11) $$u^{*}=\frac{\int_{a}^{b}u^{*}\mu (u)du}{\int_{a}^{b}\mu (u)du}$$

where $u^{*}$ is the amount of clarity, *u* is the amount of control of the output, *µ* is the affiliation function, *b* is the upper limit of the clarity value, and *a* is the lower limit of the clarity value.

### 3.2. Beetle Antennae Search

Beetle Antennae Search (BAS) represents a sophisticated and intelligent optimization algorithm introduced in 2017. This algorithm draws inspiration from biological principles, particularly the foraging behavior of the aspen insect. Unlike traditional optimization methods, BAS operates without prior knowledge of the function’s exact form or the need for gradient information, enabling efficient optimization. In contrast to other optimization algorithms such as particle swarm optimization, BAS distinguishes itself by requiring only a single individual, akin to a solitary aspen, to execute the optimization search. This characteristic significantly diminishes the computational overhead, making BAS a compelling choice for optimization tasks [9].

The principle behind the beetle antennae search algorithm is that when a beetle forages for food, it does not know the exact location of the food but rather forages because of the strength of the food’s odor. The beetle has two antennae, and if the left side receives a stronger odor pheromone than does the right side, the aspens will move to the left, and vice versa. Based on this simple principle, the beetle is able to find food efficiently. Inspired by this, people compare the function to the food of the aspens; the food smells differently at different locations in space; that is, the function has different values at different points, and the beetle finds the location of the food by collecting the maximum point of the odor value around it to solve the optimal value of the function problem [10]. The basic steps of optimization are as follows:

Step 1: For an n-dimensional spatial optimization problem, determine the left and right whisker coordinates $x_{l}$ and $x_{r}$, the center of mass coordinates *x*, the distance between the two whiskers $d_{0}$, and the step size step.

Step 2: The tenebrous ox is arbitrarily oriented, so the vectors of the left and right whiskers are also arbitrarily oriented, generating a random direction dir=rand(n,1). This dir=dir/norm(dir) is normalized to yield:

(12) $$\left\{\begin{array}{l} x_{l}=x+d_{0}\cdot dir/2\\ x_{r}=x-d_{0}\cdot dir/2 \end{array}\right.$$

Step 3: For the function to be optimized f, find the values of the left and right whiskers $F_{left}=f(x_{l}),F_{right}=f(x_{r})$, and determine the size of the two values.

(13) $$x^{t+1}=\left\{\begin{array}{l} x^{t}+step\cdot dir(x_{l}-x_{r})\;F_{left}<F_{right}\\ x^{t}-step\cdot dir(x_{l}-x_{r})\;F_{left}>F_{right} \end{array}\right.$$

where $x^{t+1}$ denotes the center of mass coordinates for *t* + 1 iterations of the beetle and step=eta·step,eta is between (0, 1).

Step 4: Determine whether the optimization accuracy is met or the maximum number of iterations is reached; if it is met, then the optimization ends; otherwise, repeat the process of steps 2 and 3.

### 3.3. Improvement of the Beetle Antennae Search Algorithm

Compared with other genetic algorithms, the beetle antennae search algorithm is characterized by individual search behavior and a simpler structure. It does not require specific knowledge of the objective function or gradient information, making it capable of achieving better convergence speed and solution accuracy in optimization tasks. However, the step size of the beetle antennae search algorithm has a more significant effect on the convergence speed of the algorithm and the strength of its ability during the search [11]. In the traditional beetle antennae search algorithm, the value of the step decay factor is a fixed value, so each iteration of the step is a fixed multiple of the previous round [12]. If the step decay is too slow, the algorithm has a strong global search capability, but the convergence speed is slow; if the step decay is too fast, the algorithm will fall into the local optimum, and the global optimal solution cannot be obtained.

Therefore, the step factor needs to be optimized, and the algorithm is improved by adopting the method of dynamic step size; in the early stage of the algorithm iteration, a larger step factor is used to improve the global search ability of the algorithm, and in the late stage of the algorithm iteration, a smaller step factor is used to improve the search accuracy of the algorithm to seek the optimal solution. The specific algorithm optimization formula is as follows:

(14) $$\mathrm{step}(t)={\mathrm{step}}_{start}-\frac{{\mathrm{step}}_{start}-{\mathrm{step}}_{end}}{T_{\max}^{2}}t^{2}$$

where: ${step}_{start}$ is the initial weight value; ${step}_{end}$ is the weight value at the end of the iteration; t is the current iteration number; and $T_{max}$ is the maximum iteration number.

According to the above formula, it can be seen that the time and step length of the algorithm are negatively correlated, and the step length is a quadratic function of time: the step length changes slowly at the beginning of the algorithm iteration, which enables the algorithm to carry out global optimization at the beginning of the iteration, avoiding falling into the local optimal solution; when the algorithm iterates to the later stage, the change in the step length is faster, which enables the algorithm to quickly converge to the optimal value after searching for optimal value domains to improve local search accuracy. The combination of these two can improve the search speed under the condition of guaranteeing accuracy. According to the above improved design, this paper proposes an improved step-size beetle antennae search algorithm, and the specific flow of the algorithm is as follows:

Step 1: Initialize the parameters. Initialize the population, the maximum number of iterations, the initial step size and so on.

Step 2: Based on the current position, the beetle starts to search.

Step 3: Update the position of the left and right tentacles of the beetle, calculate the fitness value, and update the position of the beetle. If the fitness value of the left tentacle is small, the beetle moves to the left; if the converse, it moves to the right.

Step 4: Calculate the evaluation function ITAE to update the global optimal beetle position.

Step 5: Determine whether the maximum number of iterations is reached; if not satisfied then perform step 6; otherwise, perform step 7.

Step 6: Update the step factor and perform the next round of iteration.

Step 7: Output the optimal value, the algorithm ends.

**Figure 5:**
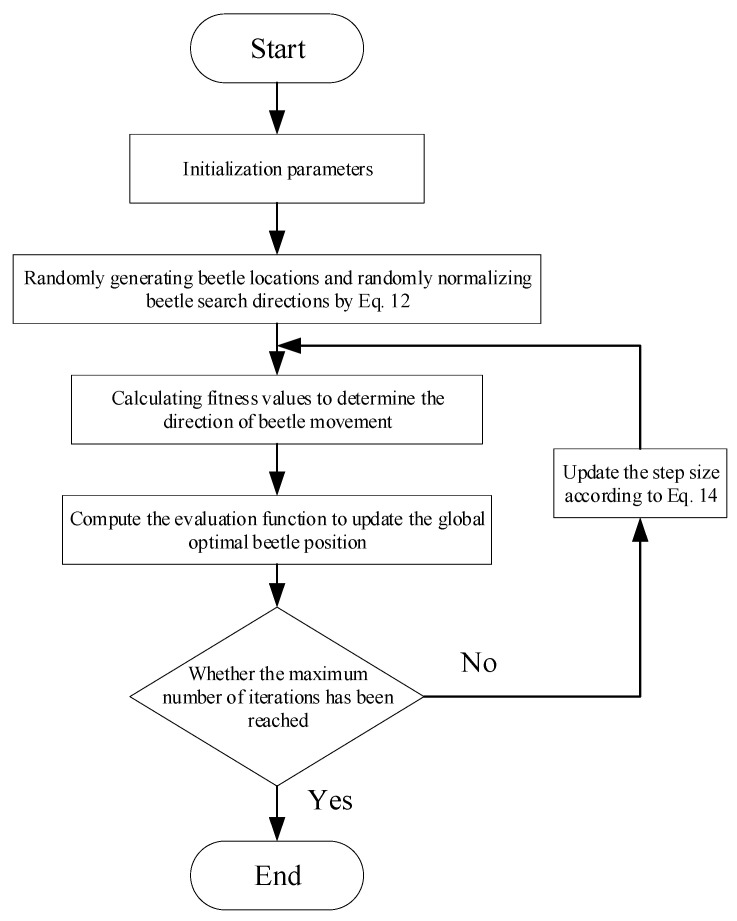
Flowchart of the improved beetle antennae search algorithm.

The flowchart of algorithm improvement is shown in Figure 5:

### 3.4. Improved Beetle Antennae Search Algorithm for Optimizing Fuzzy PID Parameters

Appropriate proportional, differential, and integral coefficients play a vital role in enhancing the error performance and response time of a control system. By employing a fuzzy algorithm to handle the system’s error and its rate of change, the optimal proportional, differential, and integral coefficients can be determined more effectively [13].

In this paper, we use the improved BAS algorithm to optimize the fuzzy PID controller in terms of the error $k_{e}$, error rate of change $k_{ec}$, proportional coefficients $k_{p}$, integral coefficients $k_{i},$ and differential coefficients $k_{d}$, integrating the five parameters together as a “beetle” in the given search space (the beetle according to the left and right whisker position of the adaptability of the value of the adjustment of the update of the next iteration of position), until the specific optimization steps meet the conditions. Until the iteration conditions are met, the specific optimization steps are as follows:

The initial population size is defined, and the maximum number of iterations is set.

A five-dimensional space is constructed where each dimension represents the error, error change rate, proportional coefficient, integral coefficient, and differential coefficient.

Define the fitness function. This paper utilizes the integral of time multiplied by the absolute error (ITAE) criterion as the fitness function, which serves as a performance index for the control system. A smaller ITAE indicates better performance. The ITAE expression is as follows:

(15) $$J(ITAE)=\int_{0}^{t}t\left|e(t)\right|dt$$

The beetle antennae search optimization algorithm is executed, and the optimized values of the five parameters ($k_{e}$, $k_{ec}$, $k_{p}$, $k_{i}$, and $k_{d}$) are extracted as the optimal solution for the given problem.

The Simulink simulation model of the gate motor control system is utilized by invoking the “sim” function within the program. The optimized parameters are integrated into the system, and the optimized control waveform is generated for the control system.

**Figure 6:**
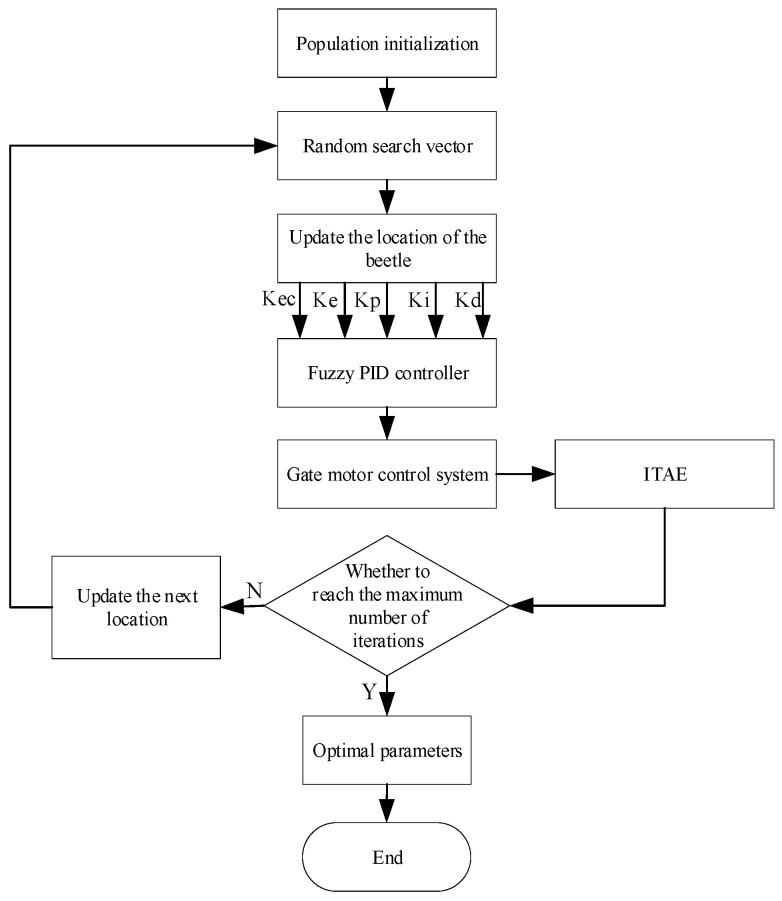
Flowchart of fuzzy PID parameter optimization by the beetle antennae search algorithm.

The optimization process for the fuzzy PID parameters using the beetle antennae search algorithm is illustrated in Figure 6.

## 4. Control System Simulation Analysis

### 4.1. PID Control System Simulation

Identifying the controlled object is a fundamental aspect of control system simulation. In this paper, the focus is on the gate system, whose operational component is primarily driven by the motor [14]. Table 2 provides an overview of the motor parameters that are essential for the experiment.

Based on the control model and system parameters, the closed-loop feedback control transfer function for the gate motor control system is represented as follows:

(16) $$G(s)=\frac{10.65s^{2}+8.493s}{8.341s^{4}+10.51s^{3}+235.5s^{2}+70.73s}$$

**Figure 7:**
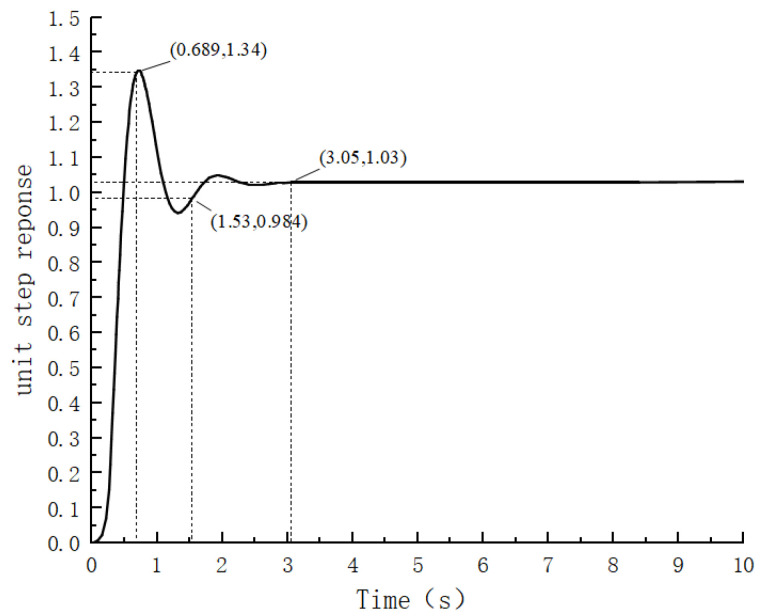
PID control simulation waveforms.

According to the PID control model of the gate motor control system established in this paper, the amplitude of the input step signal is set to 1, and the parameters of the PID controller are adjusted to analyze the waveform of the system output. The simulation waveform is shown in Figure 7.

From Figure 7, when the control system steady-state error is within 3%, the response time is 1.53 s, and the overshoot is 0.34. When this system is stabilized at a value of 1.03, it can be concluded that a certain error exists in the steady-state value over the input value of 0.03, which is not ideal for its control effect.

### 4.2. Fuzzy PID Control System Simulation

Fuzzy PID control is an extension of the traditional PID algorithm. It utilizes both the error (e) and the rate of change of error (ec) as inputs to the fuzzy system. Through fuzzy logic rules and reasoning, the fuzzy PID controller adjusts the PID parameters, allowing for adaptive rectification of the PID parameters. This approach theoretically enables fuzzy PID control to achieve a certain level of adaptability and robustness in controlling systems [15].

**Figure 8:**
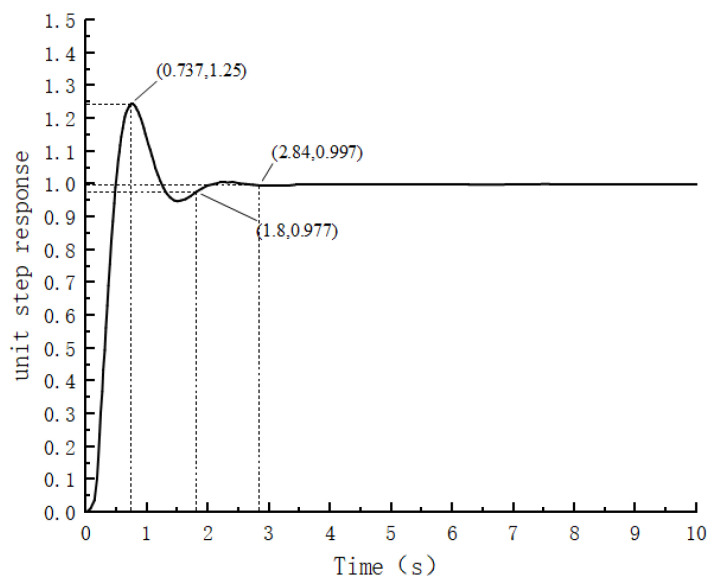
Simulated waveform of Fuzzy PID control.

The simulation results of the fuzzy PID control of the control system in this paper are shown in Figure 8.

As depicted in Figure 8, upon stabilizing the system error within a 3% range, the response time of the fuzzy PID controller is observed to be 1.8 s, indicating a 0.27 s increase compared to traditional PID control. Additionally, the overshoot of the fuzzy PID system is 0.25, showing a reduction of 0.09 units compared to traditional PID control. However, even after optimizing the PID parameters using the fuzzy algorithm, although the steady-state error is reduced, it remains nonnegligible. This suggests that solely relying on a fuzzy controller for adjusting PID control system parameters may not yield ideal results. While the overshoot has been mitigated, it comes at the cost of increased system response time, indicating a trade-off that prevents achieving the best of both worlds.

### 4.3. Simulation of a Fuzzy PID Control System Based on an Improved Beetle Antennae Search Algorithm

**Figure 9:**
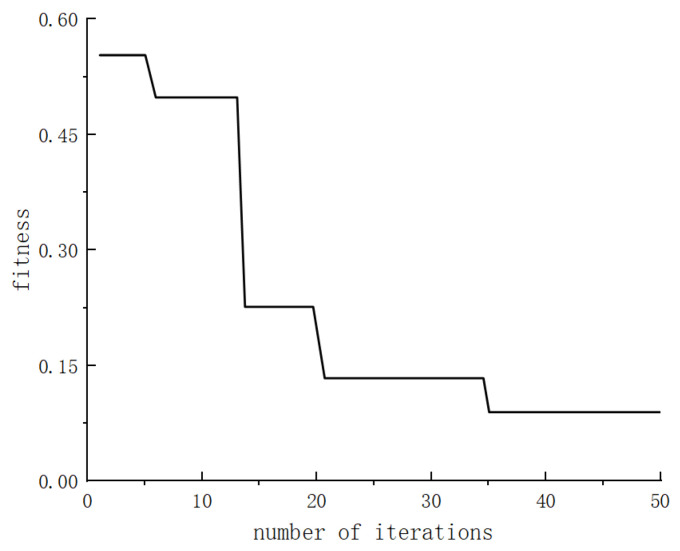
Optimal individual fitness curve.

**Figure 10:**
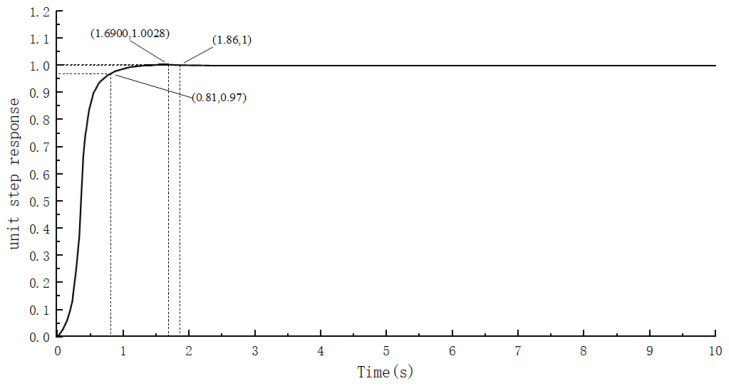
Fuzzy PID simulation waveform of the improved beetle antennae search algorithm.

By fine-tuning the step size within the beetle antennae search algorithm, this study enhances the traditional antennae algorithm to achieve optimal solutions for the system while ensuring swift optimization of the search processes. This paper establishes a simulation model of the control system by integrating the design process for optimizing fuzzy PID parameters with the beetle antennae search algorithm. System characteristics are assessed using the ITAE performance index. Figure 9 and Figure 10 illustrate the optimal individual fitness and its corresponding iterative process control system curves.

Figure 10 clearly shows that the control system, following the optimization of the fuzzy PID parameters through the enhanced beetle antennae search algorithm, achieves notable performance metrics. With the system error stabilized at 3% or less, the control system exhibits a remarkable response time of 0.81 s and a minimal overshoot of 0.0028. Moreover, the steady-state error, subsequent to tuning the PID parameters via the beetle antennae search algorithm, aligns closely with the input step signal.

As shown in Table 3, the FPID controller optimized by the beetle antennae search algorithm has a faster response time and less overshooting than do the PID and FPID controllers, and is more accurate at controlling the steady-state values.

## 5. Results and Discussion

Given that real-world control engineering often involves complex higher-order systems, the transfer function of the controlled object in this paper is depicted by Equation (9), which represents a higher-order unstable system. Through the simulation experiments conducted in the preceding sections on traditional PID and fuzzy PID control, it becomes evident that relying solely on traditional methods for integrating PID parameters yields subpar results. The response time, overshoot magnitude, and time to reach steady state are all comparatively larger, underscoring the limitations of traditional approaches in addressing the complexities of higher-order systems.

In this study, we apply the traditional beetle antennae search algorithm (BSA), the improved beetle antennae search algorithm (IBAS), the particle swarm algorithm (PSO), and the improved particle swarm algorithm (IPSO) to the parameter tuning of fuzzy PID controllers. Subsequently, a comprehensive analysis is conducted to compare and evaluate the outcomes derived from each algorithmic approach [16,17,18].

The parameters of the beetle antennae search algorithm are set as follows: the population size is 30, the number of iterations is 100, the distance between two whiskers $d_{0}$ = 2, the step size is 1, and the minimum fitness value is 0.1.

The parameters of the improved beetle antennae search algorithm are set as follows: the population size is 30, the number of iterations is 100, the distance between the two whiskers is $d_{0}$ = 2, the initial step size is 1, the minimum fitness value is 0.1, and the step size is adjusted by using Equation (14).

The parameters of the particle swarm algorithm are set as follows: the particle swarm size is 80, the number of iterations is 100, the learning factor is $c_{1}$ = $c_{1}$ = 2, the inertia weight coefficients are ω=0.8, the particle velocities are $v_{max}=1$ and $v_{min}=-1$, and the three parameters $K_{p}$, $K_{i}$, and $K_{d}$ take values in the range of [0 100].

The parameters of the particle swarm algorithm are set as follows: the particle swarm size is 80, the number of iterations is 100, the learning factor $c_{1}$ = $c_{1}$ = 2, the inertia weight coefficients $\omega_{max}=0.8$ and $\omega_{min}=0.4$, the particle velocities $v_{max}=1$ and $v_{min}=-1$, and the three parameters $K_{p}$, $K_{i}$, and $K_{d}$ have values in the range of [0 100].

**Figure 11:**
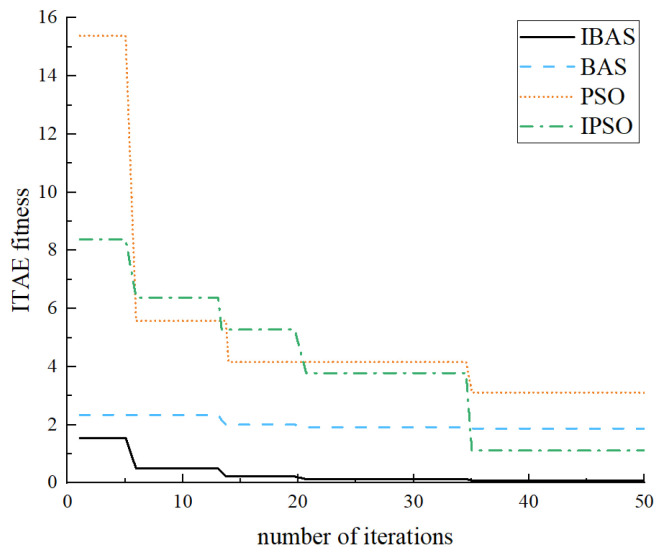
Convergence curve of the fitness function.

The convergence curves depicting the optimal individual fitness values for the performance metrics of the optimized fuzzy PID controller parameters, evaluated under the ITAE criterion, are presented in Figure 11. These curves illustrate the convergence behavior of the beetle antennae search algorithm, the improved beetle antennae search algorithm, the particle swarm algorithm, and the improved particle swarm algorithm.

Figure 11 clearly shows that the optimized beetle antennae search algorithm converges after approximately five iterations, showing superior initial fitness during the convergence phase. In contrast, the standard beetle antennae search algorithm tends to converge toward a local optimum, resulting in marginally inferior fitness compared to its improved counterpart. Comparatively, both the particle swarm algorithm and the improved particle swarm algorithm demonstrate poorer convergence performance when juxtaposed with the optimized beetle antennae search algorithm.

**Figure 12:**
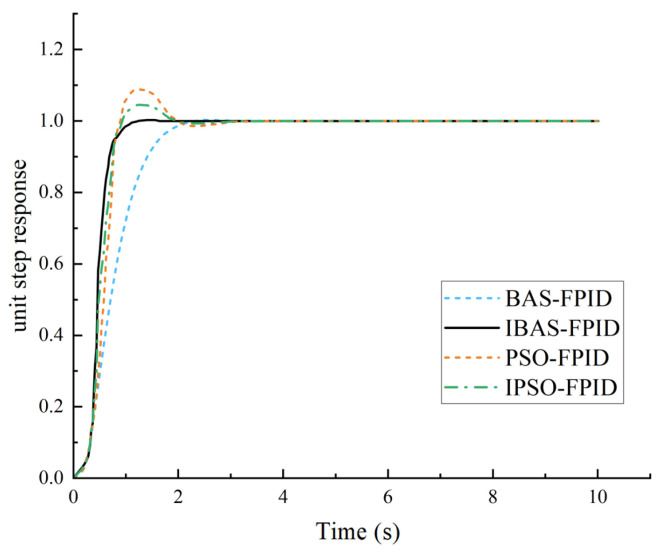
Comparison of step response curves.

The corresponding curves depicting the simulation steps of the fuzzy PID system for the four algorithms are illustrated in Figure 12.

From the experimental findings, it is apparent that the Particle Swarm Algorithm exhibits a quicker response rate. However, it demonstrates poorer performance in terms of overshoot amount and regulation time. Although the optimized particle swarm algorithm mitigates some overshooting, it still lags in regulation time. Conversely, the classical beetle antennae search algorithm shows superior control and shorter regulation time concerning overshooting. However, its response performance is comparatively subpar, necessitating a longer response time. Notably, the improved beetle antennae search algorithm outperforms all three algorithms in terms of overshoot reduction, response speed, and adjustment time. These results underscore the efficacy of the improved beetle antennae search algorithm in fine-tuning fuzzy PID parameters, particularly under the ITAE performance index.

## 6. Experimental Analysis

**Figure 13:**
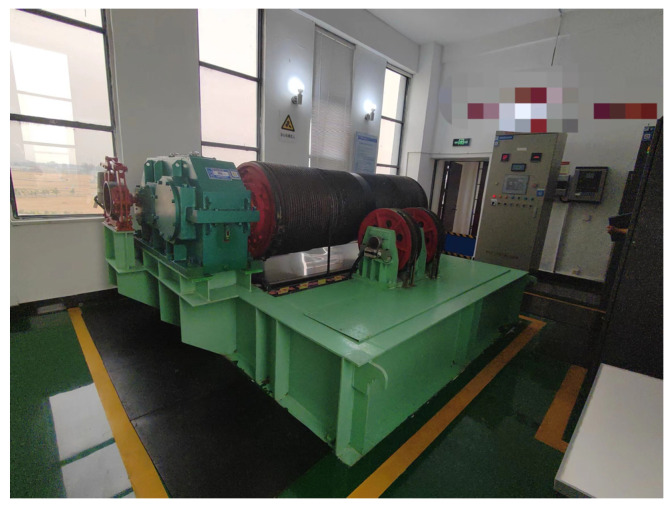
Winch opener and PLC control cabinet.

**Figure 14:**
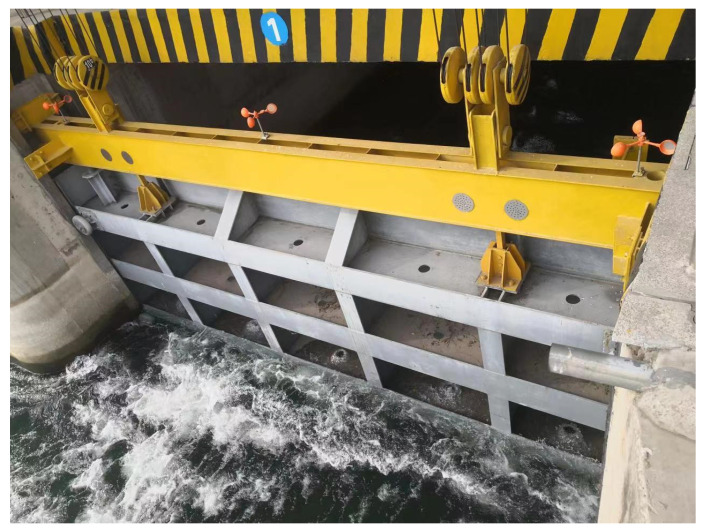
Water conservancy sluice gate.

To further verify the actual control effect of the gate control system under the improved beetle antennae search algorithm, this paper carries out a real working environment verification, and the experimental results are shown in Figure 13 and Figure 14. The experimental equipment mainly consists of a gate and a winch-type opener, programmable logic controller (PLC) control cabinet, and host computer. To verify the effect of the system under real working conditions, the rated power of the motor used is 30 kW, the rated pull force is 150 kN, the motor speed range is 885 r/min, and the gate load is 11.2 tons.

The specific working principle of the experiment is as follows: the MATLAB/Simulink environment in the host computer is opened, the motor control model of the improved fuzzy PID controller optimized by the beetle antennae search algorithm is compiled, and then the TIA portal software is run to establish communication between the host computer and the PLC control cabinet, which can send the scheduling signals to the host computer to control motor movement after the algorithm model is processed.

**Figure 15:**
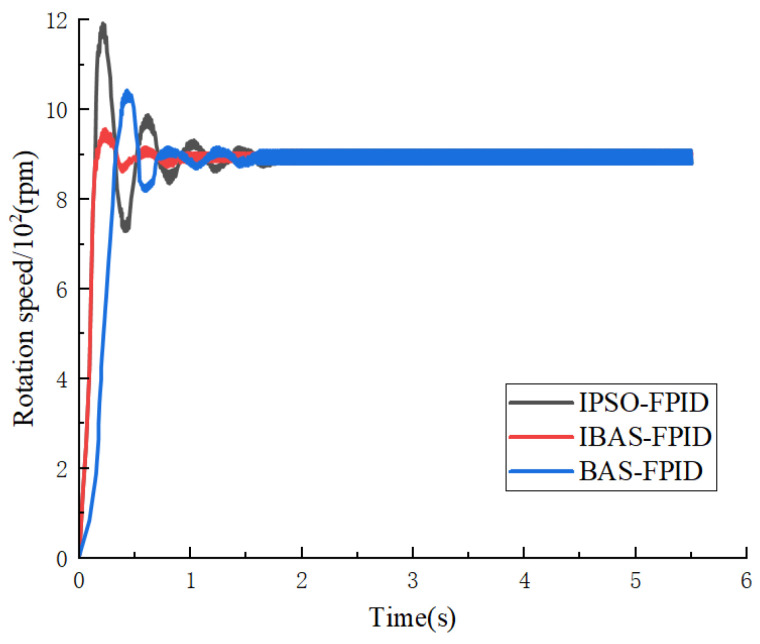
Comparison of motor speed response under different algorithmic controls.

To verify that the improved beetle antennae search algorithm optimizes the fuzzy PID controller effect under real working conditions, whether it has the same superior effect on the processing of step signals, the experimental model setup is consistent with the setup of the simulation experiment set up above, and the rotational speed of the motor of the opener is monitored to determine the control effect of the algorithm model. Since the PSO algorithm presented poor results in the simulation experiment session, the algorithm was eliminated in the validation phase: the IPSO, BAS, and IBAS algorithms were used for validation, and the experimental results are shown in Figure 15.

Compared with the IPSO and BAS algorithms, the optimized fuzzy PID controller with the improved beetle antennae search algorithm is able to follow the speed of the motor more accurately, and in the motor start-up phase, the fluctuation value of the motor speed estimation with the improved tensile whiskers algorithm is smaller than that of the other two algorithms, and is closer to the real speed of the motor. When the motor speed tends to stabilize, the speed following is more accurate, and the speed estimation fluctuates less with the actual value.

## 7. Conclusions

The conclusion drawn from this paper underscores the effectiveness of the improved step-size beetle antennae search algorithm in tuning fuzzy PID parameters, offering a viable scheme for hydraulic gate control studies. The optimized algorithm showed rapid convergence and superior optimization outcomes. In Fuzzy PID control, the establishment of fuzzy rules often relies on expert intuition, introducing subjectivity and limiting accessibility for those unfamiliar with fuzzy control. However, for the complex system investigated in this study, the optimization achieved by the improved step-size beetle antennae search algorithm outperforms that achieved by the traditional beetle antennae and particle swarm algorithms. Consequently, this algorithm adequately meets the control demands of complex systems, significantly enhancing the accuracy and response time of the gate control system.

## Figures and Tables

**Table 1:** Fuzzy rules.

$K_{p}/K_{i}/K_{d}$	$e_{c}$						
	NB	NM	NS	Z	PS	PM	PB
NB	PB/NB/PS	PB/NB/NS	PM/NM/NB	PM/NM/NB	PS/NS/NB	Z/Z/NM	Z/Z/PS
NM	PB/NB/PS	PB/NB/NS	PM/NM/NB	PS/NS/NM	PS/NS/NM	Z/Z/NS	NS/Z/Z
NS	PM/NB/Z	PM/NM/NS	PM/NS/NM	PS/NS/NM	Z/Z/NS	NS/PS/NS	NS/PS/Z
Z	PM/NM/Z	PM/NM/NS	PS/NS/NS	Z/Z/NS	NS/PS/NS	NM/PM/NS	NM/PM/Z
PS	PS/NM/Z	PS/NS/Z	Z/Z/Z	NS/PS/Z	NS/PS/Z	NM/PM/Z	NM/PB/Z
PM	PS/Z/PB	Z/Z/NS	NS/PS/PS	NM/PS/PS	NM/PM/PS	NM/PB/PS	NB/PB/PB
PB	Z/Z/PB	Z/Z/PM	NM/PS/PM	NM/PM/PM	NM/PM/PS	NB/PB/PS	NB/PB/PB

**Table 2:** Main structure and performance parameters of the electric motor.

Parameter	Parameter Value
Armature resistance $R_{a}$	4.8 Ω
Armature inductance $L_{a}$	21 mH
Torque coefficient $k_{m}$	46.32 N·m/A
Reverse potential coefficient $k_{e}$	55.3 V/(rad/s)
Motor moment of inertia $J_{m}$	$0.5\;Kg\cdot m^{2}$
Motor damping factor $D_{m}$	40 Nm/(rad/s)
Load moment of inertia $J_{L}$	$25\;Kg\cdot m^{2}$
Motor housing damping factor $D_{L}$	230 Nm/(rad/s)
Shaft stiffness $k_{12}$	32,000 Nm/Deg

**Table 3:** Comparison of control effects of PID, FPID and BSA-FPID.

Control Methods	Overshoot	Response Time	Steady State Value
PID	0.34	1.53	1.03
FPID	0.25	1.8	0.997
BAS-FPID	0.0028	0.81	1

## Data Availability

Data are contained within the article.
